# Comparative analysis of the complete genome of an epidemic hospital sequence type 203 clone of vancomycin-resistant *Enterococcus faecium*

**DOI:** 10.1186/1471-2164-14-595

**Published:** 2013-09-01

**Authors:** Margaret MC Lam, Torsten Seemann, Nicholas J Tobias, Honglei Chen, Volker Haring, Robert J Moore, Susan Ballard, Lindsay M Grayson, Paul DR Johnson, Benjamin P Howden, Timothy P Stinear

**Affiliations:** 1 Department of Microbiology and Immunology, University of Melbourne, Parkville 3010, Victoria, Australia; 2 Victorian Bioinformatics Consortium, Monash University, Clayton, Victoria 3800, Australia; 3 Australian Animal Health Laboratory, CSIRO, Geelong, Victoria 3220, Australia; 4 ARC Centre of Excellence in Structural and Functional Microbial Genomics, Clayton, Victoria, Australia; 5 Austin Centre for Infection Research (ACIR), Infectious Diseases Department, Austin Health, Heidelberg, Victoria 3084, Australia; 6 Department of Medicine, University of Melbourne, Heidelberg, Victoria 3084, Australia; 7 Microbiology Department, Austin Health, Heidelberg, Victoria 3084, Australia; 8 Department of Microbiology, Monash University, Clayton, Victoria 3800, Australia

**Keywords:** Vancomycin resistant enterococci, VRE, Sequence type 203, Antibiotics, Whole genome, Transposon, Nosocomial

## Abstract

**Background:**

In this report we have explored the genomic and microbiological basis for a sustained increase in bloodstream infections at a major Australian hospital caused by *Enterococcus faecium* multi-locus sequence type (ST) 203, an outbreak strain that has largely replaced a predecessor ST17 sequence type.

**Results:**

To establish a ST203 reference sequence we fully assembled and annotated the genome of Aus0085, a 2009 vancomycin-resistant *Enterococcus faecium* (VREfm) bloodstream isolate, and the first example of a completed ST203 genome. Aus0085 has a 3.2 Mb genome, comprising a 2.9 Mb circular chromosome and six circular plasmids (2 kb–130 kb). Twelve percent of the 3222 coding sequences (CDS) in Aus0085 are not present in ST17 *E. faecium* Aus0004 and ST18 *E. faecium* TX16. Extending this comparison to an additional 12 ST17 and 14 ST203 *E. faecium* hospital isolate genomes revealed only six genomic regions spanning 41 kb that were present in all ST203 and absent from all ST17 genomes. The 40 CDS have predicted functions that include ion transport, riboflavin metabolism and two phosphotransferase systems. Comparison of the vancomycin resistance-conferring Tn*1549* transposon between Aus0004 and Aus0085 revealed differences in transposon length and insertion site, and *van* locus sequence variation that correlated with a higher vancomycin MIC in Aus0085. Additional phenotype comparisons between ST17 and ST203 isolates showed that while there were no differences in biofilm-formation and killing of *Galleria mellonella*, ST203 isolates grew significantly faster and out-competed ST17 isolates in growth assays.

**Conclusions:**

Here we have fully assembled and annotated the first ST203 genome, and then characterized the genomic differences between ST17 and ST203 *E. faecium*. We also show that ST203 *E. faecium* are faster growing and can out-compete ST17 *E. faecium*. While a causal genetic basis for these phenotype differences is not provided here, this study revealed conserved genetic differences between the two clones, differences that can now be tested to explain the molecular basis for the success and emergence of ST203 *E. faecium*.

## Background

Enterococci are part of the human gastrointestinal tract microbiota but some species within the genus are also significant opportunistic nosocomial pathogens [1]. Enterococcal infections can be difficult to treat because of their intrinsic and acquired resistance to many classes of antibiotics, including what are often considered last-line agents such as vancomycin and daptomycin [2,3]. The difficulties associated with treatment, coupled with the risk of cross transmission to other patients, make enterococcal infections a significant infection control issue in hospitals [1].

Enterococci that are resistant to vancomycin are collectively referred to as vancomycin-resistant enterococci (VRE) [4,5]. Vancomycin resistance is conferred by the presence of one of nine different gene clusters, although the majority of VRE strains possess either the *vanA* or van*B* operon [6-8]. *Enterococcus faecium* and *Enterococcus faecalis* are the two enterococci most frequently causing human infections, however vancomycin resistance has become increasingly common predominately in *E. faecium,* in the past two decades [9,10]. The first reported cases of VRE occurred in Europe and the UK in the 1980s and VRE has since been reported in hospitals worldwide [11,12]. In Australia, the first known case of VRE was reported at the Austin Hospital in 1994 [13]. VRE has since emerged Australia-wide and a number of Australian hospitals have experienced VRE outbreaks, predominantly involving colonization in the clinical environment [14]. In contrast to the United States where *vanA*-type resistance predominates, *vanB*-type vancomycin resistant *E. faecium* are the most important cause of VRE infections in Australia [15].

Molecular epidemiology of *E. faecium* has been based primarily on pulsed field gel electrophoresis (PFGE) and multi-locus sequence typing (MLST) [16,17]. MLST has revealed the emergence of related lineages called clonal complex 17 (CC17), which comprise strains associated with hospital infections across at least five continents [18]. Many of these hospital strains have acquired resistance to ampicillin and quinolones, and their genomes contain a high number of mobile genetic elements that distinguish them from community-acquired and non-pathogenic strains [18-20].

We have previously reported on a significant increase in cases of *E. faecium* bacteraemia at a major Australian Hospital. MLST of the *E. faecium* isolates collected from bacteraemic patients over a 12-year period revealed a replacement of CC17 sequence type 17 (ST17) strains, with emergent CC17 ST203 isolates [14]. A survey evaluating the incidence of VRE and vancomycin-susceptible enterococci (VSE) in Australia from 2005 to 2010, conducted by the Australian Group on Antimicrobial Resistance (AGAR) in collaboration with participating microbiology labs, showed a marked increase in VRE rates throughout Australia. Reflecting the very same trends observed at Austin Health, the majority of these Australian VRE strains not only possessed the *vanB* gene but were also ST203 [21]. In our initial study we compared partially assembled genome sequences for representative ST17 (Aus0004) and ST203 (Aus0085) VRE isolates and showed 500 kb of unique DNA differentiating the two [14]. We also recently established and analysed the complete genome sequence of *E. faecium* ST17 Aus0004 as a basis for a more thorough genome comparison [22].

In this current study, genome and phenotype comparisons were performed to explore in more detail the differences between 13 ST17 and 15 ST203 *E. faecium* isolates to help explain the emergence of ST203 and its success as an opportunistic hospital pathogen. We fully assembled and annotated the genome of *E. faecium* ST203 Aus0085, the first complete ST203 reference sequence, and compared it with the recently fully assembled and annotated genomes of ST17 Aus0004 and ST18 isolate TX16 [22,23]. Growth rates and biofilm formation were also measured for a collection of ST17 and ST203 isolates, and virulence was assessed in the Greater Wax Moth (*Galleria mellonella*) invertebrate model.

## Results

### *Enterococcus faecium* Aus00085 genome summary

**Figure 1:**
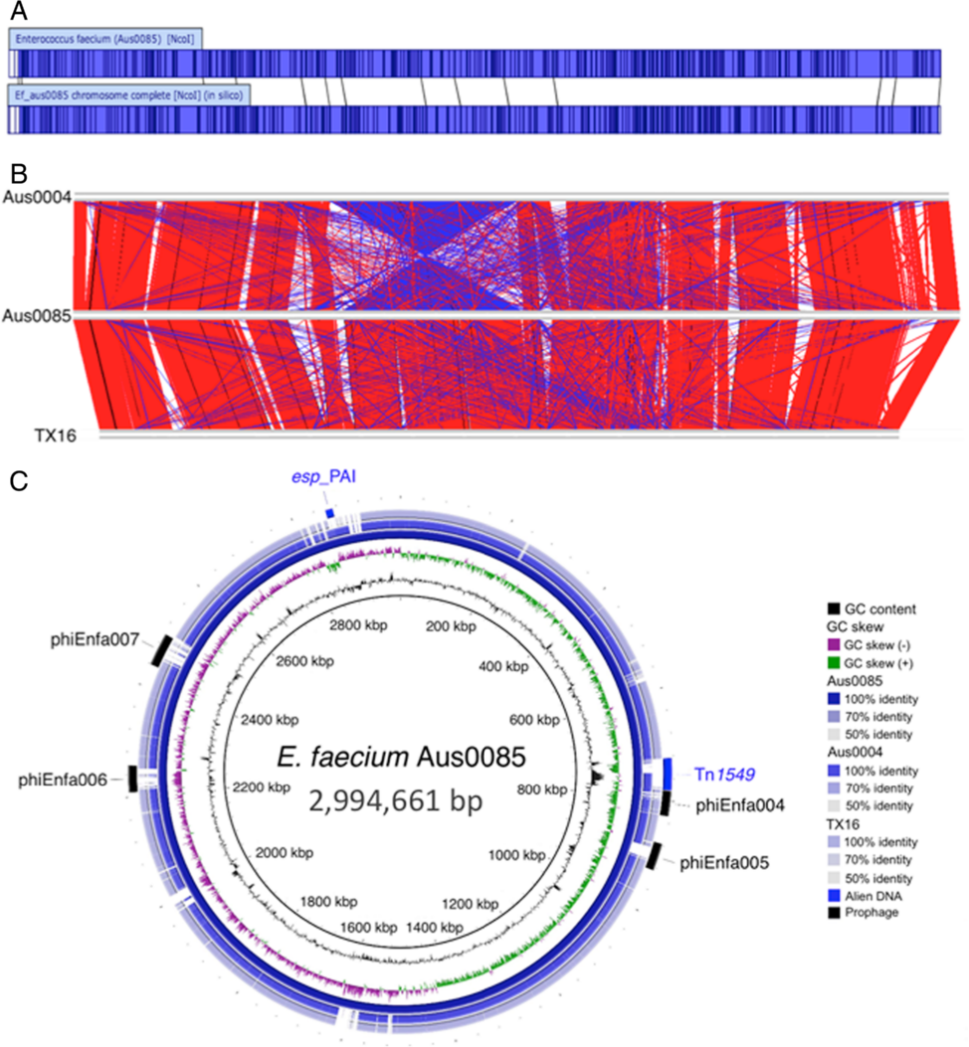
**Comparative analysis of the complete genome sequence of *E. faecium *Aus0085. (A)** Alignment of *E. faecium* Aus0085 NcoI optical map with an *in silico*-generated NcoI map of the final chromosome assembly, demonstrating the accuracy of the assembly. **(B)** Alignment of the chromosomes from *E. faecium* strains Aus0004, Aus0085 and TX16, generated using the Artemis Comparison Tool. The gray bands located at the top, middle and bottom represent the forward and reverse DNA strands for the chromosome sequences belonging to Aus0004, Aus0085 and TX16 respectively. The red lines correspond to regions of similarity between two chromosomes. The blue lines correspond to regions of similarity between two chromosomes in differing orientations. White regions are those that are unique to one strain and absent in the other. **(C)** Circular map of ST203 Aus0085 showing DNA-DNA comparisons against ST17 Aus0004 and ST18 TX16. Track identification, moving outwards, is as follows: G + C content, GC skew, *E. faecium* Aus0085, *E. faecium* Aus0004, *E. faecium* TX16, and finally the location of prophage regions, Tn*1549* transposon and the *esp*-pathogenicity island.

The genome of *E. faecium* Aus0085 is comprised of a single circular chromosome (2,994,661 bp) with a GC content of 38.2%, 2,922 protein-coding DNA sequences (CDS), 75 tRNA genes, and 6 rRNA operons. Comparisons of an *in silico* NcoI map of the fully-assembled Aus0085 chromosome sequence against the Aus0085 NcoI optical map confirmed the accuracy of the chromosome assembly (Figure 1A). Aus0085 also harbors six circular plasmids, Aus0085_p1, Aus0085_p2, Aus0085_p3, Aus0085_p4, Aus0085_p5 and Aus0085_p6, with lengths of 130,716 bp, 67,314 bp, 31,004 bp, 9,319 bp, 4,072 bp, 2,189 bp respectively.

### Comparative genomics of Aus0085 *E. faecium*

The chromosome features for ST203 *E. faecium* Aus0085 were compared with ST17 *E. faecium* Aus0004 and another recently closed *E. faecium* genome, TX16 (Table 1). Chromosome alignments indicated significant conservation of genome architecture between the three isolates. The large chromosome inversion seen in Aus0004 is not present in Aus0085 and TX16 (Figure 1B). DNA comparisons and ortholog cluster analysis showed that *E. faecium* Aus0085 possesses at least 8 chromosomal segments spanning approximately 224 kb that are not present in both Aus0004 and TX16, and an additional four regions spanning 133 kb that are absent in Aus0004 but present in TX16. These Aus0085 ‘specific’ regions include four distinct prophage, and loci involved in metabolic functions such as carbohydrate utilization and metal ion transport. A complete list of Aus0085 genes not present in Aus0004 or TX16 is provided see (Additional file 1: Table S1).

**Table 1:** **Genome comparisons between *E. faecium *strains Aus0004 (ST17), Aus0085 (ST203) and TX16**

	**ST17 Aus0004**	**ST203 Aus0085**	**ST18 TX16**
Chromosome size (G + C%)	2,955,294 bp (38.4%)	2,994,661 bp (38.2%)	2,698,137 bp (38.15%)
Plasmids (size, G + C%)	Aus0004_p1 (56,520 bp, 35.4%)	Aus0085_p1 (130,716 bp, 34.9%)	pDO1 (36,262 bp, 36.51%)
	Aus0004_p2 (4,119 bp, 36.8%)	Aus0085_p2 (67,314 bp, 35.2%)	pDO2 (66,247 bp, 34.38%)
	Aus0004_p3 (3,847 bp, 39.0%)	Aus0085_p3 (31,004 bp, 34.6%)	pDO3 (251,926 bp, 35.97%)
		Aus0085_p4 (9,319 bp, 30.9%)	
		Aus0085_p5 (4,072 bp, 36.2%)	
		Aus0085_p6 (2,189 bp, 38.9%)	
CDS	2,932	3,222	3,114
rRNA operons	6	6	6
tRNA	47	75	64

**Figure 2:**
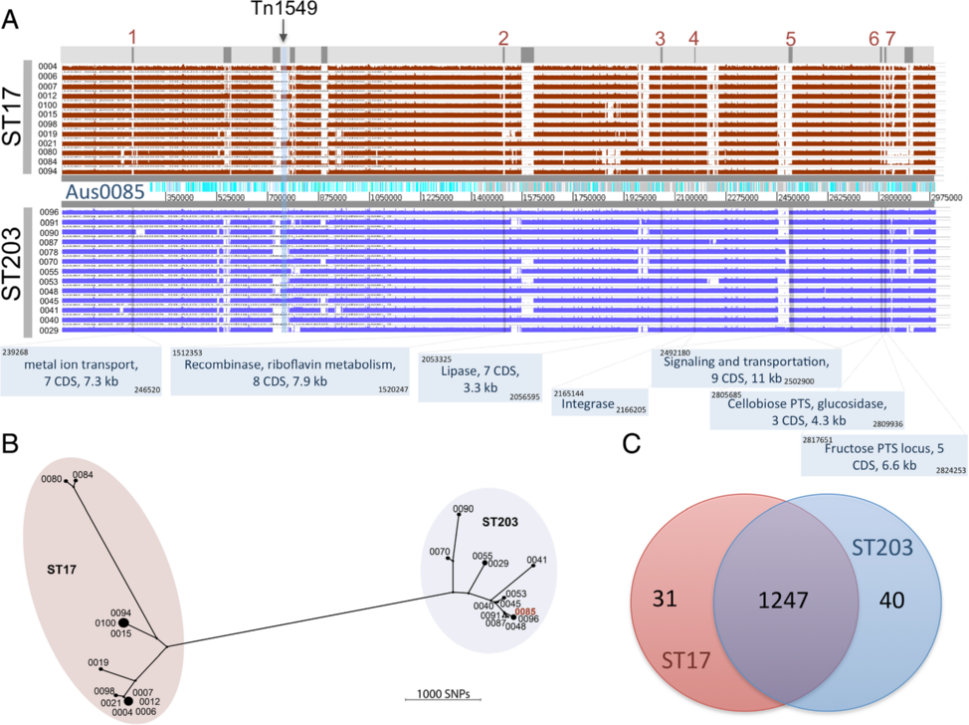
**Comparative multi-isolate genome analysis. (A)** Read-mapping outputs from whole genome sequencing of an additional 12 ST17 and 14 ST203 isolates against the completely-assembled chromosome sequence for Aus0085. The six genomic regions and integrase gene present in all ST203 isolates and absent in all ST17 isolates are highlighted and numbered. The remaining unnumbered highlighted areas correspond to regions in the Aus0085 genome that are not present in Aus0004. **(B)** SNP-based neighbor-joining tree, showing the relationship between the ST17 and ST203 isolates. **(C)** Venn diagram depicting the shared genes present in all the ST17 and ST203 strains and the specific genes that were present in all ST17 and absent from all ST203 isolates, and vice-versa.

To refine this comparative analysis, whole genome sequence reads obtained from an additional 12 ST17 and 14 ST203 previously described hospital isolates were mapped to the Aus0085 chromosome. Seven distinct regions of difference were identified, spanning 41 kb and 40 genes found in all 15 ST203 isolates but absent from the ST17 collection (Figure 2A). The majority of these genes encoded proteins with functions related to metabolism including riboflavin biosynthesis (Ef_aus0085_01495), gene regulation (Ef_aus0085_00243, Ef_aus0085_01493, Ef_aus0085_02507, Ef_aus0085_02509), metal ion transport (Ef_aus0085_00246) and phosphotransferase systems (Ef_aus0085_02768, Ef_aus0085_02779) (Figure 2A and Additional file 2: Table S2).

Read-mapping also permitted delineation of a core genome, revealing 2,217,189 bases, (Aus0085 genome: 75% core and 25 % accessory) that were present in all 28 *E. faecium* genomes. Pairwise comparisons of the Aus0085 core genome sequence with the core genomes of the other isolates uncovered 9865 variable nucleotide positions (including substitutions and indels). A distance matrix was prepared and a robust phylogeny was inferred by both split decomposition analysis and neighbor-joining methods that highlighted the substantial nucleotide differences between ST203 and ST17 isolates (Figure 2B). A reticulated phylogeny was observed by split decomposition, indicating likely recombination among this collection of *E. faecium* (data not shown). These substantial clone-specific differences were then confirmed by independent *in silico* analysis. De novo assembly with gene and protein prediction of the 26 *E. faecium* genomes (Table 2), followed by ortholog clustering that also included Aus0085 and Aus0004 based on MLST category, confirmed the same 40 ST203-specific genes as identified by read-mapping (Figure 2C, Additional file 2: Table S2). The two MLST groups shared 1247 genes, while there were 31 genes specific to the ST17 isolates (Figure 2C, Additional file 2: Table S2).

**Table 2:** ***E. faecium *strains used in this study**

**Strain**	**Year of isolation**	**MLST sequence type**	***van *type**
Aus0004	1998	ST17	*vanB*
Aus0006	1999	ST17	-*
Aus0007	1999	ST17	*-*
Aus0012	2000	ST17	*vanB*
Aus0100	2000	ST17	*-*
Aus0015	2002	ST17	*-*
Aus0098	2003	ST17	*-*
Aus0019	2004	ST17	*-*
Aus0021	2004	ST17	*vanB*
Aus0026	2005	ST17	*-*
Aus0029	2005	ST203	*-*
Aus0040	2006	ST203	*-*
Aus0041	2006	ST203	*-*
Aus0043	2006	ST203	*-*
Aus0045	2007	ST203	*vanB*
Aus0048	2007	ST203	*vanB*
Aus0053	2007	ST203	*-*
Aus0055	2007	ST203	*vanB*
Aus0070	2008	ST203	*vanB*
Aus0078	2008	ST203	*-*
Aus0080	2008	ST17	*-*
Aus0084	2009	ST17	*vanB*
Aus0085	2009	ST203	*vanB*
Aus0087	2009	ST203	*vanB*
Aus0090	2009	ST203	*vanB*
Aus0091	2009	ST203	*vanB*
Aus0096	2009	ST203	*vanB*
Aus0094	2009	ST17	*-*

*indicates strains that are vancomycin-susceptible isolates, which therefore do not possess the *van* operon.

### The Aus0085 genome is largely comprised of mobile genetic elements

Like Aus0004 and TX16, a large proportion of the genome for Aus0085 is comprised of mobile genetic elements. A total of 91 insertion sequence elements (ISEs), representing twenty different types of ISEs, were identified. The copy numbers of the ISEs varied from 1 to 24. The most frequently occurring ISE differed between Aus0004 (ISEf1, 13 copies) and Aus0085 (ISEfa5, 24 copies). Four distinct prophage were identified in Aus0085 including, phiEnfa004 (782514–827419), phiEnfa005 (876860–925798), phiEnfa006 (2208334–2256786) and phiEnfa007 (2439241–2497696). The Aus0085 prophage are distinct to the prophage regions present in Aus0004 and TX16, although as demonstrated in Figure 1C, many of the CDS that make up phiEnfa004 also appear in the other two genomes, which suggests this prophage is reasonably conserved. Interestingly, the two CRISPR-associated proteins present in the prophages phiEnfa001 and phiEnfa002 in Aus0004 are not present in Aus0085, and no other CRISPR loci were detected in Aus0085.

### The Tn*1549-*like *vanB*-containing transposon in Aus0085 is distinct from Aus0004

**Figure 3:**
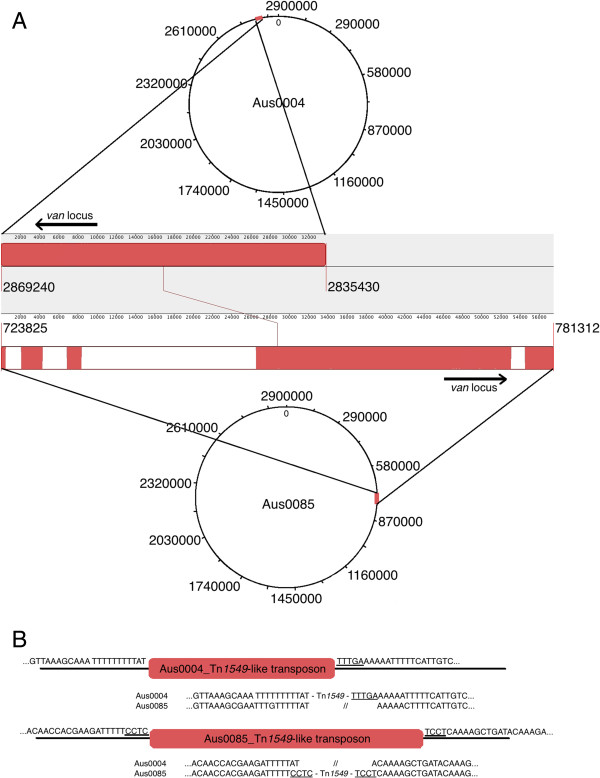
**Analysis of Tn1549. (A)** Chromosomal location and alignment of Tn1549 from *E. faecium* isolates Aus0004 (nucleotide positions 2835430–2869240) and Aus0085 (nucleotide positions 723825–781312, reverse orientation). Compared to Tn1549 in Aus0004, the element in *E. faecium* Aus0085 is 23.7 kb longer. White regions correspond to the unique 23.7 kb sequence. The red regions correspond to matches between the two transposons. **(B)** Transposon insertion site comparisons showing the left-hand side and right-hand side flanking regions and donor (coupling) sequences.

A single copy of a Tn*1549*-like transposon, encoding *vanB*-type vancomycin resistance, was present in the Aus0085 chromosome, inserted within a signal peptidase I gene (EFAU085_00682) between nucleotide positions 723825–781312, which is different from the insertion site in Aus0004 (Figure 1C). Comparisons of Tn*1549* between Aus0004 and Aus0085 revealed a conserved 30 kb core (Figure 3A). However, Tn*1549* in Aus0085 is significantly larger, containing an additional 23.7 kb with 20 CDS that appear to encode another conjugative transfer system (Table 3). When Tn1549 excises from its chromosomal location, it can take 4–6 bp of flanking sequence from the donor DNA that is then incorporated in the recipient at the site of insertion [24]. Different donor sequences (also called coupling sequences) were observed between Aus0004 and Aus00085, which together with the different form of Tn*1549* indicate a different source of this transposon for each isolate (Figure 3B). The *van* locus in Aus0085 was differentiated from the Aus0004 version by 2 non-conservative amino acid substitutions that may impact function located within the histidine kinase domain of VanS (E251G) and the N-terminal substrate-binding domain of VanB (C101F). Interestingly, the vancomycin minimal inhibitory concentration (MIC) for Aus0004 was 12 mg/L compared with 256 mg/L for Aus0085.

**Table 3:** **Unique CDS in Tn1549-like transposon region in *E. faecium *Aus0085**

**Locus tag in Aus0085**	**Nucleotide position**	**CDS, predicted product**
EFAU085_00684	724423 to 725631	IS110, transposase
EFAU085_00689	728728 to 730545	HNH endonuclease domain protein
EFAU085_00692	732291 to 732638	Hypothetical protein
EFAU085_00693	732653 to 732934	Hypothetical protein
EFAU085_00694	733193 to 733975	RNase II stability modulator
EFAU085_00695	734148 to 735866	Na+/Pi- cotransporter
EFAU085_00696	735868 to 736722	Phosphate binding protein
EFAU085_00697	736688 to 736801	Hypothetical protein
EFAU085_00698	737419 to 737718	Hypothetical protein
EFAU085_00699	737742 to 738152	Single strand binding protein
EFAU085_00700	738299 to 738505	Putative transcriptional regulator
EFAU085_00701	738852 to 739373	TDP-fucosamine acetyltransferase
EFAU085_00702	739431 to 739856	Acetyltransferase, GNAT family
EFAU085_00703	740299 to 740481	Hypothetical protein
EFAU085_00704	740594 to 742105	Hypothetical protein
EFAU085_00705	742428 to 742652	Hypothetical protein
EFAU085_00706	742645 to 743211	Hypothetical protein
EFAU085_00707	743199 to 743429	Hypothetical protein
EFAU085_00708	743630 to 743995	Hypothetical protein
EFAU085_00709	743995 to 745722	Conjugal transfer relaxase TraA
EFAU085_00710	745761 to 747668	Hypothetical protein
EFAU085_00711	747933 to 748049	Hypothetical protein
EFAU085_00712	748353 to 748535	Hypothetical protein
EFAU085_00713	748562 to 750259	Resolvase family site-specific recombinase
EFAU085_00737	776918 to 777724	ISEnfa3, transposase
EFAU085_00738	777721 to 728257	ISEnfa3, transposase

### Additional plasmids in Aus0085 contribute to the Aus0085-specific genome

Aus0085 harbors six plasmids, compared to three plasmids in Aus0004. The increased number of plasmids adds an additional 180 kb to the Aus0085 genome, with plasmid sizes ranging from 2.2 kb to 130.7 kb. Table 4 contains a summary of the predicted functions of plasmid CDS. Comparisons of the plasmid sequences in Aus0085 against the genome sequences from other enterococci show that the majority of sequence and CDS in the Aus0085 plasmids are also present in plasmids from *E. faecium* (including Aus0004)*, E. faecalis* and other enterococcal species as well as other species from other genera such as *Staphylococcus* and *Streptococcus*. Plasmid comparisons between Aus0004 and Aus0085 reveal that Aus0085_p2 is highly related (99% sequence identity) to Aus0004_p1, a 56.5 kb plasmid present in Aus0004, although Aus0085_p2 is 10.8 kb longer. Additionally, Aus0085_p5 is closely related to Aus0004_p3. The smallest plasmid Aus0085_p6 (2.2 kb), which has no other CDS other than that encoding the replication initiation protein, possesses three regions (nucleotide positions 1–938, 1182–1608 and 1982–2189) that share significant sequence identity (80%, 92% and 85% respectively) to the cryptic plasmid pRI1 [25].

**Table 4:** **Description of plasmids present in *E. faecium *strain Aus0085**

	**Aus0085_p1**	**Aus0085_p2**	**Aus0085_p3**	**Aus0085_p4**	**Aus0085_p5**	**Aus0085_p6**
**Length**	130,716 bp	67,314 bp	31,004 bp	9,319 bp	4,072 bp	2,189 bp
**GC content**	34.9%	35.2%	34.6%	30.9%	36.2%	38.9%
**Rep family**	2	17	2	Unique	14	14
**No. of CDS**	162	78	42	12	5	1
**Comments**	*F.O.I: genes related to lactose metabolism (p1096 to p1105), citrate metabolism (p1074 to p1084)	Similar to Aus0004_p1.	F.O.I: toxin-antitoxin system (p3009 and p3010)	F.O.I: bacteriocin, lactococcin 972 family	Similar to Aus0004_p3	Comprised of only a rep protein, similar to cryptic plasmid pRI1
			*aphA*, *sat4*, *aadE* aminoglycoside resistance (p3013, p3015 and p3016)			
	Toxin-antitoxin sytem (p1087 to p1090)					
			Lincosamide nucleotidyltransferase			

*F.O.I features of interest.

A total of 99 CDS, which represents one-third of the CDS that makes up the 245 kb plasmid sequence in Aus0085, are not present in Aus0004, and some of these genes are candidates to explain the growth and antimicrobial susceptibility differences between ST17 and ST203 isolates. With the exception of EFAU085_p2057, which encodes a hypothetical protein that is found only in Aus0085, all the other Aus0085 CDS that do not appear in Aus0004 are present in the genomes belonging to other *E. faecium* isolates or other enterococcal species. Among the plasmid CDS that have been assigned a function, several encode important features that enhance the survival of Aus0085. Notably, the *aadE-sat4-aphA* gene cluster, reported in a number of multidrug-resistant *E. faecium* and *E. faecalis* strains, is present on plasmid Aus0085_p3. The genes in the *aadE-sat4-aphA* cluster confer resistance to three different antibiotics, streptomycin, streptothricin and kanamycin, respectively [26]. However, *sat4* is disrupted by an ISE (EFAU085_p3014) and is therefore unlikely to be functional. The *aadE* and *aphA* genes are intact. Streptomycin MICs differed as predicted between Aus0004 and Aus0085, with values of 48 mg/L and 1024 mg/L respectively.

Other Aus0085 plasmid CDS that encode important features that are absent in Aus0004 include a PTS-lactose uptake system (*lacG, lacE, lacF, lacD2, lacC, lacB, lacA*) on Aus0085_p1, a bacterial extracellular solute-binding protein on Aus0085_p1 and additional toxin-antitoxin systems on plasmids Aus0085_p1 and Aus0085_p3. Interestingly, Aus0085_p1 possesses a citrate lyase complex (*oadHDB-citCDEFX-oadA-citMG*), which has been previously described in various Firmicutes including *E. faecium* and *E. faecalis*[27,28]. However, this locus may be non-functional as *citC* and *citG* are predicted pseudogenes. Additionally, the other auxiliary genes required for producing an active citrate lyase appear to be absent from Aus0085_p1.

### CDS associated with virulence

The same putative enterococcal virulence factors identified in Aus0004 were also present in Aus0085. While the sequences of the genes encoding hemolysin (EFAU085_01000) and collagen-binding adhesin (*acm*; EFAU085_02356) were nearly identical to the corresponding genes in Aus0004 (99-100% identity). The enterococcal surface protein (*esp*) encodes a 1733 aa protein with a likely role in colonization [29,30]. The Aus0085 *esp* gene (EFAU085_2821) has an additional 264 bp representing an extra copy of one of its 3′ tandem repeats compared to the Aus0004 ortholog.

Bacteriocins are small protein pore-forming toxins produced by certain bacteria that can prevent the growth of closely related strains [31,32]. We therefore compared the bacteriocin gene content of Aus0085 with Aus0004 to see if additional bacterioicin-like genes might explain the emergence of Aus0085. However genome comparisons showed no novel bacteriocins in Aus0085 and both isolates shared the same complement of three bacteriocin genes (>99% nt identity between each clone), although their genome location varied. For example a gene encoding a lactococcin 972-like protein is present on Aus0085 plasmid P4 (EFAU085_p4005) but on the chromosome in Aus0004 (EFAU004_00292).

### *E. faecium* ST203 isolates grow more quickly than ST17

**Figure 4:**
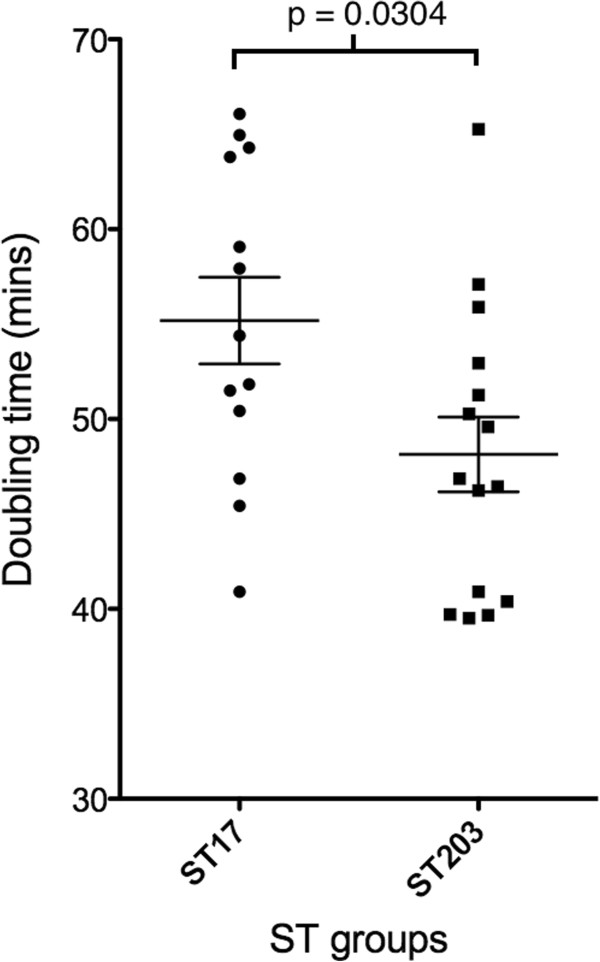
**Doubling times calculated from the growth curves of *E. faecium *strains.** The doubling times, in minutes, from early- to late- exponential phase were determined for each of the *E. faecium* strains. The values were plotted according to ST. The ST203 strains appear to grow at a faster rate than the ST17 strains and statistical analysis performed using the two-tailed Mann Whitney test demonstrated that the difference was statistically significant (p = 0.0304).

To determine whether there were any differences in growth rates between different isolates, which might be linked to the additional metabolic genes or the lactose metabolism pathway identified in the genome for ST203-representative strain Aus0085, we conducted growth curve experiments to compare doubling times between the ST17 and ST203 strains. Exponential phase doubling times were calculated and a significant difference (p = 0.0304) was observed between ST17 (average: 55.186 min, SD: 8.216 min) and ST203 isolates (average: 48.136 min, SD: 7.611 min) (Figure 4). The doubling times for the two sequenced strains were not different; Aus0004 possessed an average doubling time of 66.1 minutes while average doubling time for Aus008 was 65.3 minutes.

### *E. faecium* ST203 Aus0085 and Aus0090 out-competes ST17

**Figure 5:**
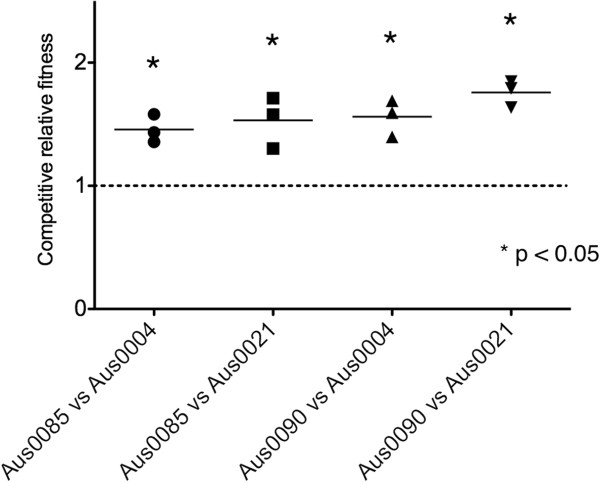
**Relative competitive fitness of ST203 isolates Aus0085 and Aus0090 compared with ST17 isolates Aus0004 or Aus0021.** Grown in the presence of ST17 strains, all ST203 strains outcompeted the ST17 strains in mixed cultures over 24 hours, possessing a competitive relative fitness index value significantly greater than 1.

We next took advantage of the high-level gentamicin resistance of Aus0085 and Aus0090 and performed co-culture experiments to assess whether these representative ST203 isolates could outcompete ST17 isolates. As indicated by the relative competitive fitness index (RCFI) (Figure 5), both ST203 isolates out-competed the ST17 isolates, Aus0004 and Aus0021, by approximately the same factor. Statistical analysis showed all comparisons were significantly higher than the threshold RCFI value of 1. Aus0004, Aus0021 and Aus0085 possessed similar doubling times of 66.068, 64.282 and 65.341 minutes respectively. However, compared to the other strains, Aus0004 took, on average, two hours longer to reach stationary phase, and Aus0090 had a significantly faster doubling time of 52.942 minutes.

### Biofilm formation is not an *E. faecium* clone-specific trait

**Figure 6:**
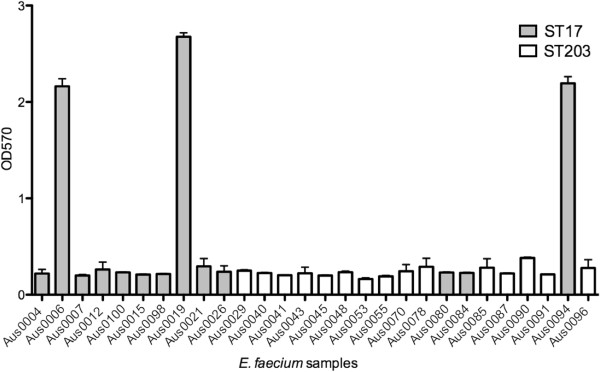
**24-hour biofilm assays, performed in TSB supplemented with 1% glucose.** OD570nm readings were measured for each strain to determine the amount of biofilm formed. Isolates Aus0006, Aus0019 and Aus0094 exhibited significantly enhanced biofilm production compared to the other isolates (p < 0.001).

The ability of *E. faecium* to form biofilms is thought to be an important phenotype with respect to virulence traits such as colonization, environmental persistence and antibiotic resistance [33-35]. We therefore performed biofilm assays to determine whether a difference in biofilm formation abilities distinguished the two ST clones. Three *E. faecium* ST17 strains Aus0006, Aus0019 and Aus0094 showed enhanced biofilm-forming capacity compared to the other isolates (p < 0.05) but no relationship with sequence type was observed (Figure 6).

### Virulence is a heterogeneous phenotype in both the ST17 and ST203 strains

**Figure 7:**
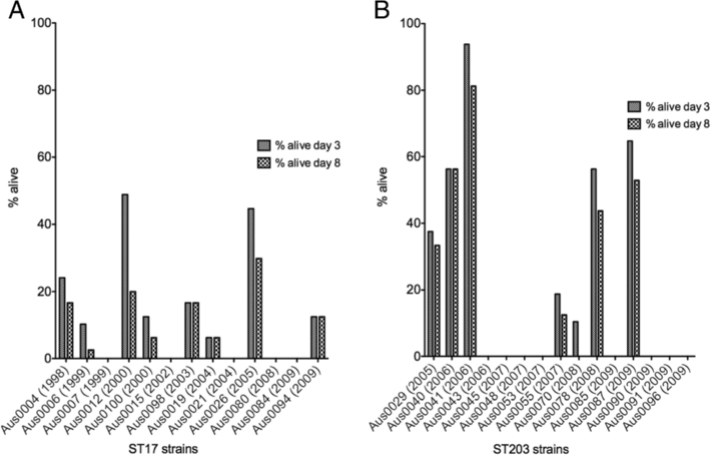
***Galleria mellonella *survival.** Percentage of survival of larvae inoculated with a **(A)** ST17 *E. faecium* strain or **(B)** ST203 *E. faecium* strain at day 3 and day 8. Both clonal groups possessed virulent strains that resulted in the death of all larvae by day 3 and avirulent strains that did not kill all the larvae by day 8.

We examined survival rates of *G. mellonella* larvae after inoculation with ST17 and ST203 *E. faecium*. The Kaplan Meier plots were analysed to compare virulence between isolates see (Additional file 3: Figure S1 and Additional file 4: Figure S2 for the individual Kaplan Meier survival plots generated for each of the ST17 and ST203 isolates, respectively). Based on observations made during the experiments, isolates were considered virulent if all larvae within a group died within three days of inoculation. Virulence varied independent of sequence type (Figure 7). However, excluding Aus0087, which exhibited small colony variant morphology, the ST203 strains isolated from 2009 onwards, killed *G. mellonella* larvae within three days. Larvae inoculated with small colony variant strains Aus0041, Aus0078 and Aus0087 exhibited increased survival rates. The number of bacteria present in the larvae at the time of death was also quantified, with values ranging from 2.2 × 10^6^ CFU/ml to 4.25 × 10^8^ CFU/ml. Substantial variations were observed in CFU, even in larvae that had been inoculated with the same isolate, suggesting that differences in growth between different sequence types does not explain the rate at which the larvae are killed.

## Discussion

Nosocomial outbreaks of *E. faecium* are now dominated by ST203 in Australia, and this ST has also been responsible for nosocomial outbreaks in Germany and China [36,37]. However, surprisingly little is known about this group of clones or why it emerged almost simultaneously in several hospitals and Australian states. Additionally, the apparent shift from ST17 *E. faecium* to ST203 observed at the Austin Hospital is reflected in a previous study that examined the population structure of *E. faecium*. Hospital isolates can be categorized into two groups (BAP2-1 and BAP3-3) [38], with ST17 isolates belonging to BAP3-3 and ST203 isolates belonging to BAP2-1. These observations correlate with the trends observed at the Austin Hospital and in hospitals around Australia, where strains belonging to BAP3-3 dominated from 1990 to 2004, and have been replaced with BAP2-1 isolates from 2005 onwards [14].

In this report, we have fully assembled and annotated the genome of strain Aus0085. To our knowledge, this is the first complete ST203 *E. faecium* genome sequence*.* Comparisons with the only two other complete *E. faecium* genomes, ST17 Aus0004 and ST18 TX16, were performed to understand the genetic basis behind the emergence and increase in ST203 VRE. Comparative analysis revealed 502 kb of sequence present only in Aus0085. These regions of difference spanned eight chromosomal segments in Aus0085 (including four distinct prophage) and four of the six plasmids harbored by this isolate. Genome comparisons were extended to a collection of ST17 and ST203 isolates and this reduced the amount of ST203 ‘unique’ DNA to six chromosome regions. Inferred functions from genes within these regions suggest additional metabolic capabilities for the ST203 strains, particularly in carbohydrate utilization and metal ion transport. Additional ST203-unique metabolic genes include a riboflavin biosynthesis gene, dihydrofolate reductase and a fructose phosphotransferase system (PTS). PTS are numerous in both gram-positive and negative bacteria, and facilitate the transport, uptake and metabolism of carbohydrates. A recent functional study showed that deletion of *E. faecium ptsD*, which encodes a sugar-specific membrane-associated EIID subunit of a PTS gene cluster common to hospital isolates and absent in commensal isolates, compromises the ability of *E. faecium* to colonise the intestinal tract of mice under antibiotic treatment [39]. The results from this study and our observations here, showing two putative PTS loci present in all ST203 but absent from ST17 (Figure 2A), underscore the likely important contributions of PTS systems (loci beginning at Ef_aus0085_02768 and Ef_aus0085_02778), as well as other metabolic functions, in defining the success of a strain within a host and possibly the clinical environment.

Approximately 24% of the Aus0085-specific genes (relative to Aus0004 and TX16) are found on the six plasmids, highlighting the importance of these elements in shaping the Aus0085 genome and the potential for their transmission to other clinical *E. faecium* isolates. Aus0085_p1 possesses genes with predicted lactose functions that might allow for utilization of alternative carbohydrate sources. Enterococci in humans and other animals colonize the gastrointestinal tract and consequently, they compete with the gut microbiota for nutrients and resources [1,40,41]. The presence of additional metabolism genes might therefore confer a survival and growth advantage to ST203 in this environment. A functional genomic study recently conducted investigating carbohydrate metabolism in *Streptococcus pneumoniae* revealed a 20-minute increase in doubling time in a lactose PTS system mutant, suggesting that the additional lactose utilization pathway in Aus0085 might similarly promote the growth of ST203 [42]. Plasmid Aus0085_p3 possesses an intact version of the *aadE* and *aphA* genes, which confer resistance to streptomycin and kanamycin respectively. Streptomycin susceptibility testing revealed a 20-fold increase in the MIC of Aus0085 (1024 mg/L) compared to Aus0004 (48 mg/L). The *aphA* kanamycin resistance gene also confers resistance against gentamicin, which likely explains the high-level gentamicin resistance observed in Aus0085. Clinical isolates that exhibit high levels of resistance against gentamicin are often treated using streptomycin. Resistance against streptomycin in Aus0085, probably conferred by *aadE*, is therefore a concern.

The genome comparisons also revealed significant insights into the acquisition of the vancomycin resistance-conferring transposon in the representative genomes. Several transposons that are responsible for mediating *vanB*-type resistance against vancomycin have previously been described in enterococci: Tn*1547*, Tn*1549* and Tn*5382*. The 27 kb Tn*5382* shares high sequence homology to the 34 kb Tn*1549*[43-45]. Previous studies investigating the acquisition of vancomycin resistance have provided some clues to the sources and mode of transposon transmission, but there remains much to be determined [46-48]. While vancomycin resistance in both Aus0004 and Aus0085 is conferred by the *van* operon associated with a Tn*1549*-like conjugative transposon, thorough transposon comparisons revealed substantial differences between the transposon length, sequence and site of insertion. The additional genes with a possible role in conjugation may consequently affect transposon transmission and spread. These variations show that Aus0004 and Aus0085 acquired their transposons from different donors. Non-synonymous nucleotide changes within the *van* locus coincide with the extent of vancomycin resistance in Aus0085, with antimicrobial susceptibility testing showing a 20-fold increase in the vancomycin MIC for Aus0085 compared to Aus0004. The E251G substitution within the histidine kinase domain of VanS and the C101F change within the N-terminal substrate-binding domain of VanB are both candidates for further exploration to test their role in the increased resistance to vancomycin displayed by Aus0085.

The differences between the transposons in the ST17 and ST203 isolates not only relate to function but also provide important insight into the transmission and sources for acquisition of transposons in clinical *E. faecium* strains. The location of the *van* locus on mobile elements permits spread via horizontal gene transfer as well as clonal spread. It is important to establish the exact mechanisms that lead to the acquisition of antibiotic resistance, particularly in hospital-adapted strains, in order to understand the emergence of VRE and to prevent the spread of resistance to VSE or other bacterial species. While both Aus0004 and Aus0085 possess transposon insertion sites that differ from those previously described as preferred integration sites [47] (Figure 3), further analysis may reveal common insertion sites that distinguish the ST17 and ST203 isolates. Identifying these sources for transposon and vancomycin-resistance acquisition may potentially lead to diagnostic differences that can be exploited prevent VRE infection through the elimination of the sources.

We have also characterized some of the phenotypic differences between ST17 and ST203 *E. faecium*. The isolates we selected to study came from a previously described ST203 outbreak, which reported the displacement of resident ST17 *E. faecium* VRE with ST203. Given the significant number of additional metabolic genes in Aus0085, experiments assessing the rate of growth were performed to determine whether the additional genes yielded improved growth. Comparisons of 13 ST17 and 15 ST203 isolates highlighted significantly increased growth rates and competitive fitness for ST203 that may be factors contributing to the emergence of this clone in our hospital. Notably, Aus0004 possessed a prolonged lag phase compared to the other strains, and the delay in reaching exponential phase is likely to be due to its abnormal GC skew pattern arising from imbalanced replichores [22].

ST17 and ST203 comparisons of virulence using *G. mellonella* larvae demonstrated a range of virulence phenotypes in larvae inoculated with isolates from either clonal group. A potentially interesting difference was observed with the ST203 strains isolated from 2009 onwards, killing *G. mellonella* faster than earlier isolates of either sequence type. Genome comparisons however revealed few differences between the known virulence factors in Aus0004 and Aus0085. Both genomes contained genes encoding virulence factors such as hemolysin and enterococcal surface protein (*esp*). Esp appears very important for the ability of *E. faecium* to colonize the host [30,49-51]. Variations in its length, such as observed here, may alter function and explain the difference in *G. mellonella* virulence we observed between Aus0004 and Aus0085. Variation in Esp expression [30], as well as the potential of using the apparent variability of *esp* to discriminate between different ST17 and ST203 strains, were not explored here and remain to be investigated. We currently are trying to understand global differences in gene expression between Aus0004 and Aus0085 using RNAseq and the completed reference genomes.

## Conclusions

The extent of variability between the three *E. faecium* genomes compared here highlights the significant roles played by mobile genetic elements in shaping the evolution of this species. The additional 502 kb in ST203 Aus0085 with genes that likely confer additional metabolic functions and antimicrobial resistances, help explain the emergence of this clone. These observations coincide with the, on average, faster growth and increased competitiveness of ST203 compared to ST17. While this study has provided several insights into differences that distinguish the ST17 from ST203 *E. faecium* strains, differences in the expression of particular genes also require investigation. In this respect, the completed Aus0085 genome represents an important resource as a reference for read-mapping applications such asRNAseq for exploring the *E. faecium* transcriptome, or for high-resolution molecular epidemiology to better understand the factors behind the emergence of this opportunistic nosocomial pathogen.

## Methods

### Bacterial sequences and strains

The strains used in this study were selected from isolates previously reported [14] to represent the ST17 and ST203 *E. faecium* strains collected over a 12-year period from bacteraemia patients at the Austin Hospital (Table 2). Bacterial cultures were prepared in brain heart infusion (BHI) broth unless otherwise stated.

### Antimicrobial susceptibility testing

Antimicrobial susceptibility tests determining the MICs against vancomycin and streptomycin were performed for isolates Aus0004 and Aus0085 by Macromethod Etest.

### Genome sequencing and assembly

Genomic DNA was sequenced on a 454 GS FLX instrument using a 3 kbp mate-pair library yielding 100 K reads of average length 402-bp (Roche Diagnostics, Basel, Switzerland), and on an Illumina GAIIx using a paired-end library yielding 12M reads of length 36-bp. The 454 reads were *de novo* assembled using Newbler (v2.6). The Illumina reads were used to correct errors in the Newbler scaffolds. Finishing was managed with Gap4, using combinations of PCR, primer walking and sequencing of selected clones from a 50-kb insert-size *E. coli* bacterial artificial chromosome (BAC) *E. faecium* Aus0085 clone library. Correct assembly was validated by reference to a previously described NcoI Optical Map (Opgen) [14].

### Genome analysis

Genome annotation of *E. faecium* Aus0085 was performed using the in-house tools Prokka and Wasabi (http://www.bioinformatics.net.au). Comparison of whole chromosome DNA sequences for *E. faecium* isolates Aus0004, Aus0085 and TX16 were compared using the Artemis Comparison Tool (ACT), Mauve (v2.3.1) and BLAST Ring Image Generator (BRIG) [52-55]. Mobile genetic elements such as insertion sequence elements (ISEs) and phage regions were identified using the genome annotation, ISfinder and Phast [56,57]. CRISPRfinder was used to screen for the presence of CRISPR elements [58].

### Multi-isolate comparative genomics

Through a larger, separate study examining VRE transmission, 2× 100 bp paired-end Illumina sequence reads were available for the 26 isolates listed in Table 2 (Howden et al., submitted). These reads were aligned separately to the completed Aus0085 genome using SHRiMP 2.0 [59]. Those positions in Aus0085 that were covered by at least one read from every genome defined a core genome. Read-mapping outputs were visualized in Artemis. SNPs were identified using Nesoni v0.35, a Python utility that uses the sequence reads for each genome aligned to the core genome to construct a tally of putative differences at each nucleotide position (including substitutions, insertions, and deletions). This tally was then used in a Bayesian model to decide whether a base or deletion could be called for the position, and if so, whether it differed from the reference sequence (http://www.bioinformatics.net.au). Phylogenetic analyses were performed using a distance method, based on pairwise nucleotide sequence alignments for the core genome among all isolates. Split decomposition and Neighbour-joining analysis was employed using uncorrected p distances as implemented in SplitsTree4 [60]. The reads from these 26 isolates were also subject to *de novo* assembly using Velvet (v1.2.04) [61] and annotation with Prokka. The resulting protein-coding DNA sequences (CDS) were used as for ortholog clustering analysis. Ortholog clustering was performed using derived amino acid sequences for each CDS in CD-HIT v4.6 (default parameters) [62], with the clustering requirement that specific sets have representatives in all their genomes related to that set. The sets were defined as ‘core genes present in all ST17 and ST203’, ‘ST203-specific genes’ and ‘ST17-specific genes’).

### Nucleotide accession numbers

The complete genome sequence and annotation for *E. faecium* Aus0085 has been deposited in GenBank under Bioproject ID PRJNA193299 and accession numbers CP006620-CP006626.

### Growth curves

Growth curves were generated for the *E. faecium* strains by adding a 100 μl 1:100 dilution of overnight culture, prepared in BHI broth, to individual wells in a 96-well flat-bottom plate and incubated over a 24-hour period at 37°C. Starting from time point 0, OD_600nm_ measurements were made at hourly intervals over a 24-hour period. The generation and doubling times were determined for each of the strains during a 2 hour period during exponential phase of growth according to the following formulas: Generation time (GT), in mins = (logN_t_ – logN_0_) ÷ log2, N_t_ = OD value at end of exponential period, N_0_ = OD value at beginning of exponential period, Doubling time (DT), in mins = (∆t ÷ GT) × 60, ∆t = 2 hours Growth curve experiments were repeated three times.

### Competition growth assays

For each experiment, a 1:200 dilution of an overnight culture from ST17 isolates Aus0004 or Aus0021 (low level resistance to gentamicin, MIC = 6 mg/L) and ST203 isolates Aus0085 or Aus0090 (high level resistance to gentamicin, MIC > 1024 mg/L) were separately added to 50 mL BHI broth and incubated over a 24-hour period at 37°C. Starting from time point 0, 1 mL samples were taken at hourly intervals for OD600nm measurements until a constant reading was attained and again at time point 24 h. The ratio of ST17 colonies to ST203 colonies was determined by plating 100 μl of the serial dilutions prepared from the samples on BHI agar and BHI agar with 100 mg/L gentamicin. To analyse the relative fitness of the ST203 strain compared to the ST17 strain when grown together under experimental conditions, the following formula was used to calculate the Relative Competitive Fitness Index (RCFI): RCFI = [ln(number of ST203 colonies at 24 hours ÷ number of ST203 colonies at 0 hours)] ÷ [ln(number of ST17 colonies at 24 hours ÷ number of ST17 colonies at 0 hours)] [63] Each competition growth assay experiment was repeated at least three times. Statistical significance of the differences between the sample means compared to a threshold value of 1.0 were assessed using one sample *t*-test.

### Biofilm assays

Overnight cultures were prepared using tryptic soy broth (TSB) supplemented with 1% glucose. 96-well polystyrene flat-bottom microtitre plates were coated with 200 μl 1:200 diluted *E. faecium* overnight culture in TSB with 1% glucose. Following a 24-hour stationary incubation period at 37°C, the plates were washed with phosphate buffered saline (PBS), stained with 125 μl 0.1% w/v crystal violet, and left at room temperature for 10 minutes. The plates were then washed with PBS and left to dry for a further 10 minutes before the addition of 125 μl 80:20 ethanol acetone. Absorbance measurements at OD570nm were determined for each well. Experiments were repeated an additional two times.

### *Galleria mellonella* time kill assays

The virulence of the *E. faecium* strains was assessed using a previously described *Galleria mellonella* (Greater Wax Moth) invertebrate infection model that has been utilized in several studies as an invertebrate model to study the virulence of pathogenic organisms including *Staphylococcus aureus* and *Enterococcus faecalis*[64-66]. In brief, *G. mellonella* larvae in the final instar were inoculated in groups of 16 with a particular *E. faecium* isolate. Bacterial suspensions for each isolate were prepared by washing cells with PBS that were harvested from overnight cultures. Ten microliters of the bacterial suspension, comprised of approximately 0.5–1.0 × 10^9^ CFU/mL, was injected into the hamocoel via the first right proleg of each larva. The bacterial suspensions from which the inoculums were taken were plated onto BHI agar in duplicates to check the starting cell concentration. Following injection, the larvae were incubated at 37°C and the number of dead larvae were recorded daily. The number of bacteria in the larvae at time of death was quantified. This quantification was achieved by plating serial dilutions of the hemolymph onto BHI agar. The assays were repeated in biological triplicates (three different bacterial suspensions), and control groups with larvae inoculated with PBS were included. The percentage of survival at days 3 and 8 was calculated for each strain.

### Ethics statement

This study was performed in accordance with Austin Health Human Research Ethics Committee guidelines. The use of de-identified bacterial strains described in this manuscript, and the study of the bacterial isolates and not human subjects, meant that formal Human Ethics Committee approval or Informed Patient Consent was not required.

## Abbreviations

ACT: Artemis comparison tool; AGAR: Australian group on antimicrobial resistance; BAC: Bacterial artificial chromosome; BHI: Brain heart infusion; BRIG: BLAST ring image generator; CC17: Clonal complex 17; CDS: Coding sequence(s); DT: Doubling time; GT: Generation time; ISE: Insertion sequence element; MIC: Minimal inhibitory concentration; MLST: Multi-locus sequence typing; PBS: Phosphate buffered saline; PFGE: Pulsed field gel electrophoresis; PTS: Phosphoenolpyruvate:carbohydrate phosphotransferase system(s); RCFI: Relative competitive fitness index; ST: Sequence type; ST17: Sequence type 17; ST203: Sequence type 203; TSB: Tryptic soy broth; VRE: Vancomycin-resistant enterococci; VREfm: Vancomycin-resistant *Enterococcus faecium*; VSE: Vancomycin-susceptible enterococci.

## Competing interests

The authors declare that they have no competing interests.

## Authors’ contributions

MMCL designed and performed experiments, analysed data and co-wrote the paper, TS, NJT, SB, MLG, and PDRJ analysed data, HC, VH and RJM performed experiments, BPH and TPS designed the study, analysed data and co-wrote the paper. All authors read and approved the final manuscript.

## Authors’ information

Benjamin P Howden and Timothy P Stinear are joint senior author.

## Supplementary Material

Additional file 1: Table S1 Complete list of CDS unique to Aus0085 based on comparison to Aus0004. Click here for file

Additional file 2: Table S2 ST17 and ST203 ‘unique’ genes based on comparison of 13 ST17 and 15 ST203 genome sequences. Click here for file

Additional file 3: Figure S1 Individual Kaplan Meier survival curves generated by ST17 *Enterococcus faecium* isolates in *Galleria mellonella* time-kill virulence assays. Click here for file

Additional file 4: Figure S2 Individual Kaplan Meier survival curves generated by ST203 *Enterococcus faecium* isolates in *Galleria mellonella* time-kill virulence assays. Click here for file
