# Supplementary material for: Comparative analysis of the complete genome of an epidemic hospital sequence type 203 clone of vancomycin-resistant Enterococcus faecium
Source: BMC Genomics. 2013 Sep 1;14:595. doi: 10.1186/1471-2164-14-595 (PMC3846456; doi:10.1186/1471-2164-14-595)
Supplement: Additional file 2: Table S2 — ST17 and ST203 ‘unique’ genes based on comparison of 13 ST17 and 15 ST203 genome sequences. [file 1471-2164-14-595-S2.pdf]

Additional file 2: ST17 and ST203 'unique' genes based on comparison of 13 ST17 and 15 ST203 genome sequences

31 ST17 genes:

Ef_aus0004_02128	hypothetical protein
Ef_aus0004_02129	putative phage head-tail adaptor
Ef_aus0004_02130	Phage gp6-like head-tail connectorprotein
Ef_aus0004_02131	phage major capsid protein, HK97 family
Ef_aus0004_02132	phage portal protein, HK97 family
Ef_aus0004_02134	phage terminase
Ef_aus0004_02135	phage terminase, small subunit, P27 family
Ef_aus0004_02136	hypothetical protein
Ef_aus0004_02137	hypothetical protein
Ef_aus0004_02138	Virulence-associated protein E
Ef_aus0004_02139	DNA primase/polymerase
Ef_aus0004_02140	hypothetical protein
Ef_aus0004_02141	hypothetical protein
Ef_aus0004_02142	toxin-antitoxin system, toxin component,Bro domain protein
Ef_aus0004_02143	BRO family, N-terminal domain
Ef_aus0004_02144	hypothetical protein
Ef_aus0004_02145	hypothetical protein
Ef_aus0004_02147	site-specific tyrosine recombinaseXerC-family
Ef_aus0004_02157	hypothetical protein
Ef_aus0004_02685	D-xylulose kinase
Ef_aus0004_02692	hypothetical protein
Ef_aus0004_02699	hypothetical protein
Ef_aus0004_02713	hypothetical protein
Ef_aus0004_02715	hypothetical protein
Ef_aus0004_02716	hypothetical protein
Ef_aus0004_02719	hypothetical protein
Ef_aus0004_02721	replication initiator protein A
Ef_aus0004_02722	hypothetical protein
Ef_aus0004_02723	Helix-turn-helix domain
Ef_aus0004_02724	hypothetical protein
Ef_aus0004_02725	site-specific tyrosine recombinaseXerC-family

40 ST203 genes:

Ef_aus0085_00241	integral membrane protein
Ef_aus0085_00242	hypothetical protein
Ef_aus0085_00243	TetR family dihydroxyacetone kinase regulator
Ef_aus0085_00244	acetyltransferase, GNAT family
Ef_aus0085_00245	cadmium-translocating P-type ATPase
Ef_aus0085_00246	zinc/cadmium/mercury/lead-transporting ATPase
Ef_aus0085_00247	hypothetical protein
Ef_aus0085_01491	resolvase family site-specific recombinase

Ef_aus0085_01492	helix-turn-helix protein
Ef_aus0085_01493	Bacterial transcription activator, effector binding protein
Ef_aus0085_01494	hypothetical protein
Ef_aus0085_01495	riboflavin biosynthesis protein RibD protein
Ef_aus0085_01496	hypothetical protein
Ef_aus0085_01497	hypothetical protein
Ef_aus0085_01498	dihydrofolate reductase, DfrD_2
Ef_aus0085_02033	hypothetical protein
Ef_aus0085_02034	hypothetical protein
Ef_aus0085_02035	hypothetical protein
Ef_aus0085_02036	putative phosphosugar isomerase/binding protein
Ef_aus0085_02037	ISL3 family transposase
Ef_aus0085_02038	ISL3 family transposase
Ef_aus0085_02039	ISL3 family transposase
Ef_aus0085_02149	Integrase core domain
Ef_aus0085_02500	hypothetical protein
Ef_aus0085_02501	RDD family protein
Ef_aus0085_02502	signal peptide peptidase SppA_1
Ef_aus0085_02503	hypothetical protein
Ef_aus0085_02504	ABC transporter, ATP-binding protein/permease
Ef_aus0085_02505	hypothetical protein
Ef_aus0085_02506	hypothetical protein
Ef_aus0085_02507	response regulator receiver domain protein
Ef_aus0085_02509	sensor histidine kinase
Ef_aus0085_02768	PTS system, lactose/cellobiose-specific IIC component
Ef_aus0085_02769	GntR family transcriptional regulator
Ef_aus0085_02770	6-phospho-beta-glucosidase
Ef_aus0085_02778	glycosyl hydrolase family 38 protein
Ef_aus0085_02779	PTS system, fructose-specific IIBC component
Ef_aus0085_02780	PTS system, fructose-specific IIBC component
Ef_aus0085_02781	PTS system, fructose-specific IIAcomponent
Ef_aus0085_02782	transcriptional antiterminator bglG
