# Supplementary figures and images for: Comparative analysis of the complete genome of an epidemic hospital sequence type 203 clone of vancomycin-resistant Enterococcus faecium
Source: BMC Genomics. 2013 Sep 1;14:595. doi: 10.1186/1471-2164-14-595 (PMC3846456; doi:10.1186/1471-2164-14-595)

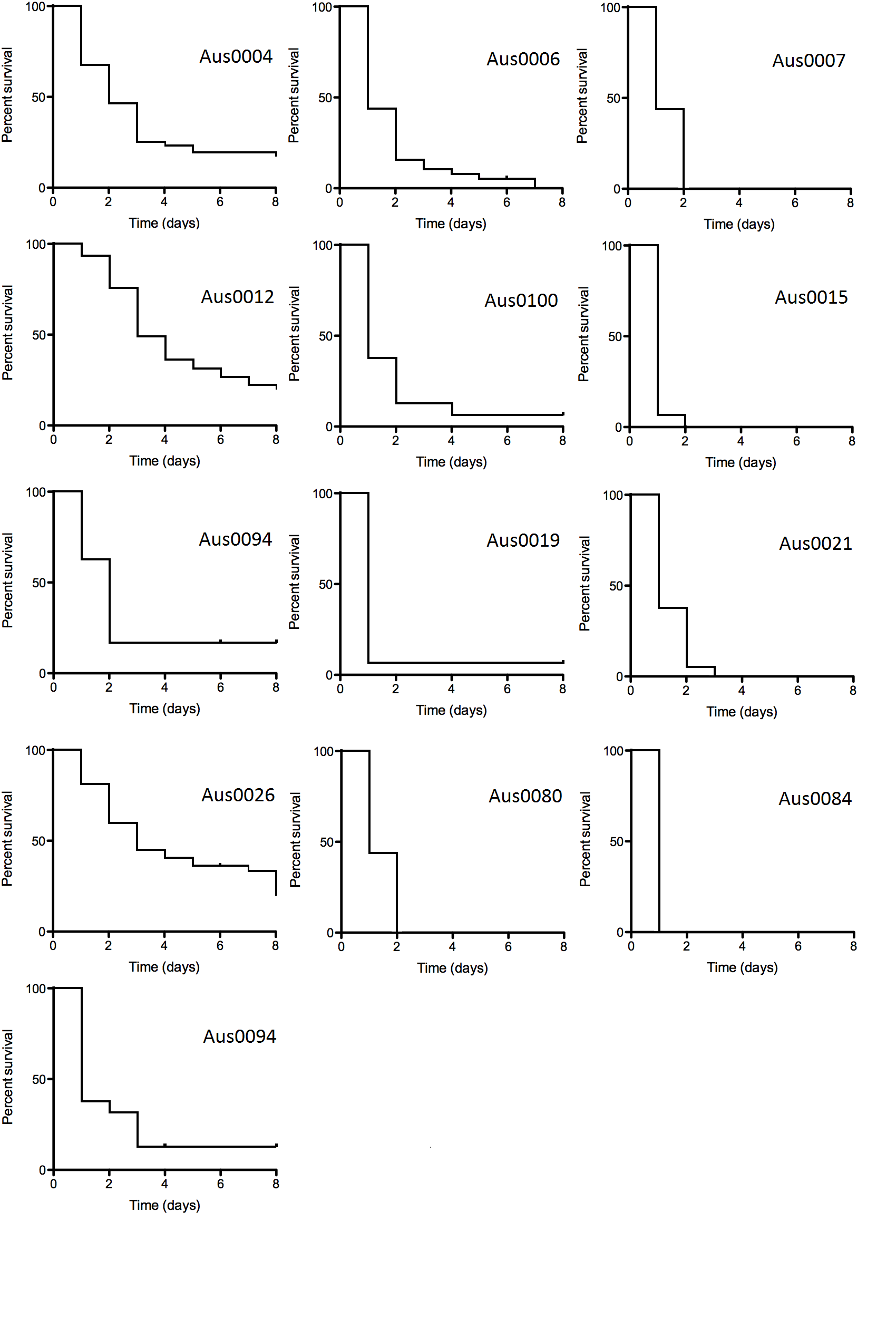

Supplement: Additional file 3: Figure S1 — Individual Kaplan Meier survival curves generated by ST17 Enterococcus faecium isolates in Galleria mellonella time-kill virulence assays. [file 1471-2164-14-595-S3.png]

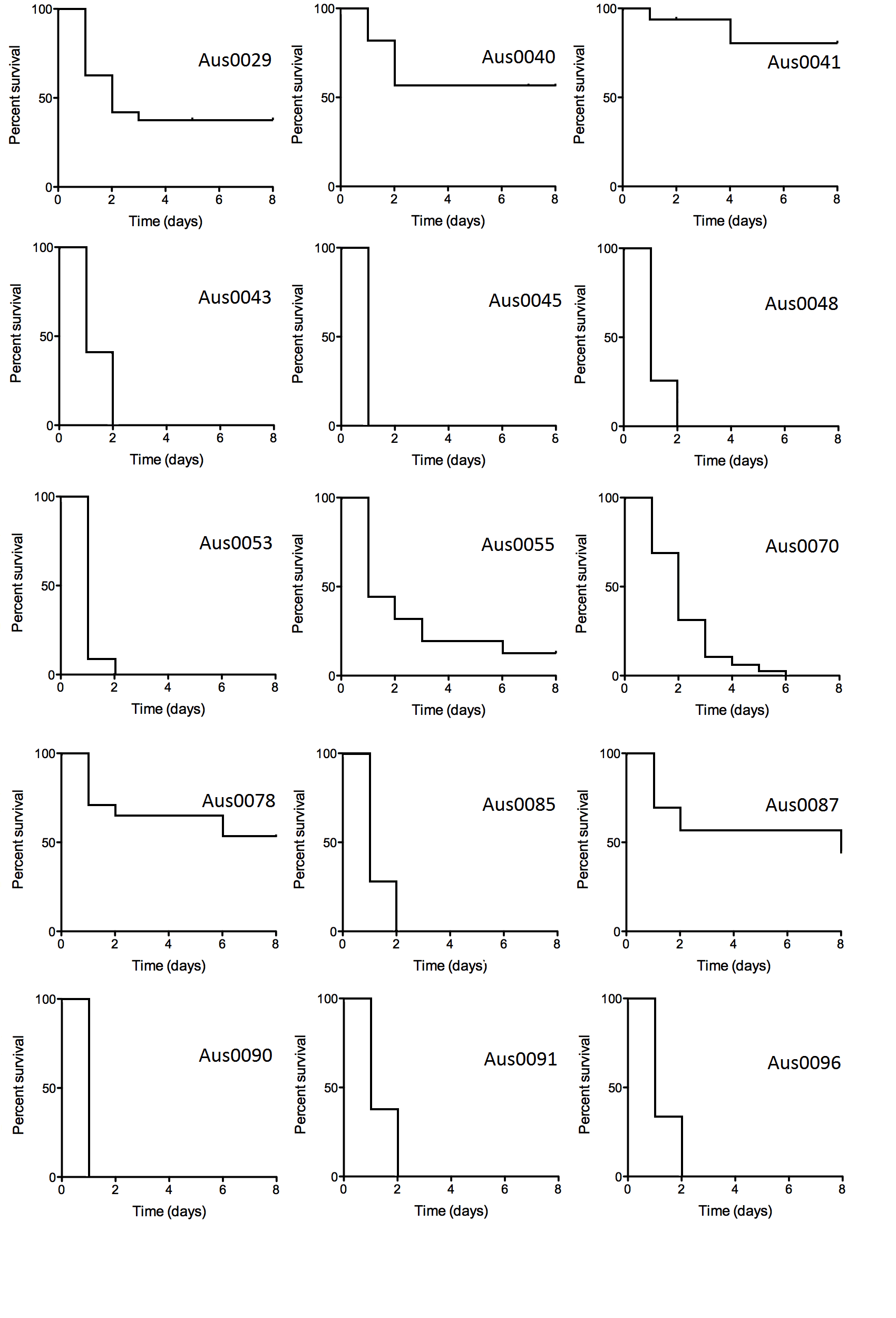

Supplement: Additional file 4: Figure S2 — Individual Kaplan Meier survival curves generated by ST203 Enterococcus faecium isolates in Galleria mellonella time-kill virulence assays. [file 1471-2164-14-595-S4.png]
